# How to personalize ovarian stimulation in clinical practice

**DOI:** 10.4274/jtgga.2017.0058

**Published:** 2017-09-01

**Authors:** Giovanna Sighinolfi, Valentina Grisendi, Antonio La Marca

**Affiliations:** 1 Department of Obstetrics and Gynecology, University of Modena and Reggio Emilia and Clinica Eugin, Modena, Italy

**Keywords:** In in vitro fertilization, controlled ovarian stimulation, individualization, anti-Müllerian hormone, antral follicle count

## Abstract

Controlled ovarian stimulation (COS) in in vitro fertilization (IVF) cycles is the starting point from which couple’s prognosis depends. Individualization in follicle-stimulating hormone (FSH) starting dose and protocol used is based on ovarian response prediction, which depends on ovarian reserve. Anti-Müllerian hormone levels and the antral follicle count are considered the most accurate and reliable markers of ovarian reserve. A literature search was performed for studies that addressed the ability of ovarian reserve markers to predict poor and high ovarian response in assisted reproductive technology cycles. According to the predicted response to ovarian stimulation (poor- normal- or high- response), it is possible to counsel couples before treatment about the prognosis, and also to individualize ovarian stimulation protocols, choosing among GnRH-agonists or antagonists for endogenous FSH suppression, and the FSH starting dose in order to decrease the risk of cycle cancellation and ovarian hyperstimulation syndrome. In this review we discuss how to choose the best COS therapy, based on ovarian reserve markers, in order to enhance chances in IVF.

## INTRODUCTION

Controlled ovarian stimulation (COS) in in vitro fertilization (IVF) cycles is the crucial point from which good oocyte retrieval and couple's prognosis depend. Several protocols have been studied in order to find the therapy that ensures the best outcomes in terms of pregnancy and live birth, minimizing iatrogenic risks, and the risk of cycle cancellation due to poor response or ovarian hyperstimulation syndrome (OHSS). In recent years, the concept of “one size fits all” has evolved into a concept of “individualization” in IVF. This should also reduce costs and the dropout rate of patients, mainly caused by the physical and psychological burden (1). Treatment individualization is based on ovarian reserve. The ovarian response to COS largely depends on a woman’s ovarian reserve, the stimulation regimen itself is a secondary factor. Serum anti-Müllerian hormone (AMH) and ultrasound antral follicle count (AFC) in particular have been shown to be the most sensitive markers. Another strategy for the individualization of treatment is based on the response in previous IVF cycles (if a previous cycle had a good performance, the same protocol can be used).

In the Italian scenario, a strong consensus exists among physicians on the importance of the prediction of ovarian response to treatment. Ovarian reserve markers are assessed in as many as 80% of women who enter IVF programs, and the majority of physicians agree that AMH and AFC are the most reliable factors for predicting ovarian response (2). The choice of therapy is a very important clinical point because of the possibility of using various kinds of drugs [gonadotrophin-releasing hormone (GnRH)-analogues or antagonists, different gonadotrophin preparations, adjuvant therapies]. Moreover, the selection of the follicle-stimulating hormone (FSH) starting dose is fundamental for IVF outcomes (3, 4, 5). In this review, we discuss how to choose the best therapy in order to improve IVF outcomes on the basis of marker-guided ovarian response predictions.

## EVIDENCE ACQUISITION

A literature search was performed for studies that addressed the ability of ovarian reserve markers to predict ovarian response in IVF cycles. A systematic search of Medline, EMBASE, Cochrane library, and Web of Science databases was conducted using the keywords, anti-Müllerian hormone, AMH, antral follicles, AFC, poor/high response, and IVF. Criteria were identified in the title and/or abstract of the publications. Additional journal articles were identified from the bibliographies of included studies as well as textbooks. Literature available up to January 2017 was included.

## EVIDENCE SYNTHESIS

### Ovarian reserve markers

We found that many ovarian reserve markers have been proposed in recent years. Serum FSH, measured on day 3-5 of the menstrual cycle, and estradiol are the most employed markers in reproductive medicine. The problem is that FSH is an indirect marker of ovarian reserve and its serum levels are out of range only when ovarian reserve is severely compromised. As a consequence, the literature reports suboptimal sensitivity and specificity for this marker in predicting ovarian response to gonadotrophins. Various cut-off values (from 10 to 15 IU/L) have been proposed for predicting poor ovarian response, but the large percentage of patients with normal values limits the usefulness of the marker.

In the last 10 years, serum AMH and ultrasound AFC have shown to measure the real ovarian follicle pool very accurately. The pool of 2 to 9 mm antral follicles measured using ultrasound when performing AFC is the same that produces AMH, so AFC and AMH are highly correlated and have the same performance in evaluating follicle quantity (6). AFC and serum AMH have shown similar predictive value for ovarian response and number of retrieved oocytes, and a better performance than other ovarian reserve markers in predicting ovarian response in IVF (7-9). A few studies found that AMH was the strongest predictor of ovarian response, whereas other studies demonstrated a stronger predictive value for AFC (10).

AMH has very little intra- and inter-cycle variability. With new recent automated assays we have repeatable and comparable dosages among laboratories. AFC is characterized by a certain intra-cycle variability and intra-and inter-observer variability due to different methodology for counting antral follicles; which class of antral follicles better correlates with the number of retrieved oocytes has yet to be demonstrated (2-5 mm, 4-6 mm or 5-10 mm). In clinical practice, 2-10 mm follicles are counted in order to obtain the AFC (11, 12). Three-dimensional (3D) automated follicular tracking decreases both intra- and inter-observer variability (13), but it requires advanced ultrasound equipment, which is not yet available everywhere. AFC and AMH are useful instruments for the individualization of ovarian stimulation regimens and for the choice of FSH starting dose in particular (2, 6). Several studies reported a linear correlation between AMH and live birth rates (14); however, the predictive value of AFC is less clear. Thus, AMH appears more useful when counselling couples about the chances of live birth after IVF.

## PREDICTIVE MODELS

Age is one of the most reliable indicators of ovarian response, but women of similar age may have wide variations in ovarian response due to different dimensions in the pool of recruitable antral follicles (15). Despite the usefulness of markers of ovarian reserve in order to individualize ovarian stimulation regimens, the literature is still lacking practical algorithms that may help physicians in choosing the right therapy and few studies proposed to individualize the treatment on a single marker, AFC or AMH.

A large randomized control trial (RCT) is ongoing with the aim of evaluating live birth rates and the cost-effectiveness of individualizing gonadotrophin starting doses on the basis of AFCs. In this study, women are categorized into groups based on AFCs and randomized to receive either individualized or standard gonadotrophin doses (16). Two studies have been published reporting the efficacy of serum AMH levels in tailoring gonadotrophin dose selection (5, 17). Nelson et al. (5) published a prospective non-randomized study that included more than 500 women undergoing IVF for whom the therapeutic protocol (standard long agonist or antagonist protocol) and FSH starting dose were chosen on the basis of basal AMH levels. The result of the personalized approach was a reduction of both the extremes of ovarian reserve with a reduction in excessive responses and cancelled cycles due to poor response (5). A retrospective study by Yates on 769 women at first IVF cycle demonstrated that an individualized, AMH-guided, controlled ovarian hyperstimulation protocol significantly improved positive clinical outcomes, reduced the incidence of complications, and reduced the financial burden associated with assisted reproduction (17). A recent pilot study compared the efficacy and safety of two algorithms, one based on AMH and the other on AFC, to determine the starting dose of recombinant FSH (rFSH) for ovarian stimulation in 348 women. Patients were assigned to receive an FSH starting dose of 150, 225 or 375 IU on the basis of pre-treatment AMH or AFC. The study reported no difference in terms of clinical pregnancy, multiple pregnancies and miscarriage rates between the two groups, but there was a statistically significant difference in ovarian response, with a major proportion of hyper responses in the AFC-tailored group (18).

## COMPLEX PREDICTIVE MODELS

Different variables are implicated in ovarian response (19, 20, 21). This concept brought about the elaboration of complex algorithms in order to better predict ovarian response and to define the right FSH starting dose. A prospective study tested a model including age, AFC, ovarian volume, Doppler ovarian score, and smoking status (19), but the complexity in measured variables did not permit wide clinical application. Another study proposed a model based on age, body mass index (BMI), day 3 serum FSH and AFC (22), which was later tested in the CONsistency in r-FSH Starting dOses for individualized tReatmenT (CONSORT) study (23). This model was not applied in clinical practice because the coefficients for computing the algorithm were not published. Moreover, the FSH starting dose calculated by the model was often lower than those proposed by clinical practice and led to iatrogenic poor responses. This was confirmed in a successive prospective study, where the CONSORT calculator was used to calculate the FSH starting dose for 197 women undergoing IVF cycles: the calculated dose was too different to the dose recommended by physicians to be applied (24).

A more recent study created a model through a retrospective analysis based on age, AFC, and day 3 serum FSH, with AFC being the most significant predictor of ovarian response (25). According to the model, in a woman aged 30 years with a normal day 3 FSH of 4 IU/L and an AFC of 16, the most appropriate gonadotrophin dose is 150 IU daily. A similar nomogram based on AMH had previously been developed by the same group and included AMH, age, and day 3 serum FSH (26). Based on the nomogram, a woman aged 30 years with FSH of 4 IU/L and AMH 4 ng/mL would require a gonadotrophin dose of 150 IU/daily. A model incorporating AMH has recently been validated retrospectively in two independent IVF centers in Italy. In both centers, the application of the nomogram resulted in more appropriate FSH starting doses compared with empirically chosen treatment. This easy-to-use algorithm could be useful in daily clinical practice for increasing the number of patients reaching optimal ovarian response (27). The AMH-based approach for COS individualization has recently been further confirmed by the results of a multicenter randomized phase-3 trial, the Evidence-based Stimulation Trial With Human rFSH in Europe and Rest of World 1 study (ESTHER-1). The study compared the efficacy and safety of a new recombinant FSH (follitropin delta) with an AMH and BMI-tailored dose with conventional recombinant FSH (follitropin alfa). The use of the new gonadotropin resulted in similar ongoing pregnancy and live birth rates, with fewer excessive and poor responses compared with the control group (28).

The prediction of ovarian response based on ovarian reserve markers may be useful for the choice of stimulation protocols and of any supplementary therapies, as discussed below.

## OVARIAN RESPONSE PREDICTION AND MANAGEMENT

After the ovarian response has been predicted, the physician has to choose the most adequate COS protocol and FSH starting dose in order to obtain an optimal oocyte retrieval. An egg collection of between 8 and 15 oocytes should guarantee the highest chances of pregnancy. Egg retrievals of less than 8 oocytes reduce pregnancy rates because of the lack of adequate numbers of good embryos to transfer. Retrievals of more than 15 oocytes may expose patients to the risk of OHSS. This means that physicians should try to obtain a moderate follicular recruitment in high responders. All protocols have demonstrated similar performance in IVF outcomes for predicted poor responders, as such the best protocol is the least stressful for the patient.

## PREDICTED POOR RESPONSE

The prognosis in IVF cycles depends on age and ovarian reserve. Assisted reproductive technology (ART) can only partially provide against the decay of fertility induced by age and reduction of ovarian reserve: pregnancy rates in women aged over 40 are less than 10%, and only slightly higher in younger women with severely reduced ovarian reserve. A low ovarian reserve translates into an inadequate response to controlled ovarian stimulation, insufficient egg retrieval, and maybe to poor oocytes and embryo quality. Poor ovarian response is defined as the retrieval of <4 oocytes following a standard IVF protocol (29). The incidence of poor ovarian response in IVF cycles ranges from 10 to 20% and the prevalence increases with advancing age.

The criteria used to identify poor ovarian responders are both anamnestic (age, shortening of the menstrual cycle, previous ovarian surgery) and clinical, based on the study of ovarian reserve. The problem of using ovarian reserve markers is in defining acceptable cut-off levels for predicted poor response. The literature reports several values for AMH and AFC in the prediction of the poor response. The variability could be explained by factors such as the small sample size of some studies and variability in the measurement of markers. According to published data, a cut-off value of AMH ranging between 0.7-1.3 ng/mL may be considered acceptable for the prediction of poor response in IVF, with sensitivity and specificity (20). AFC can be used to reliably predict ovarian response in IVF, but there is high variability in cut-off levels in the literature (10). Recent studies reported AFC cut-off values for the prediction of poor response ranging between <5 and <7 (30).

The assessment of ovarian reserve in these patients is useful during pre-treatment counseling in order to advise couples about the possibility of cycle cancellation and poor prognosis, and reduces drop-out rates. Although poor ovarian reserve is associated with poor IVF outcomes, the diagnosis of poor ovarian reserve is not acceptable as the only factor leading to direct exclusion of couples from undergoing ART treatment programs. In fact, AFC and AMH, which are the best predictive markers, have a false positive rate of 10-20%. Moreover, the accuracy of these markers is not high in the prediction of pregnancy (31), and the possibility of achieving pregnancy, especially in young women, is quite acceptable (32).

Unfortunately, there is currently insufficient evidence to recommend a particular treatment for women defined as poor responders. Treatment with a GnRH antagonist protocol instead of a GnRH agonist protocol was initially proposed for such women because it avoids the profound suppression of endogenous FSH and luteinizing hormone (LH) concentrations in the early follicular phase at the stage of follicular recruitment, giving hope of a better egg retrieval. Several trials and meta-analyses showed that the long GnRH agonist and GnRH antagonist regimens were comparable in their efficacy in terms of IVF outcomes for poor responders (33).

The few studies published on women with predicted poor response undergoing their first IVF cycle reported similar outcomes using both protocols. As shown by Nelson et al. (5), the GnRH antagonist protocol was associated with fewer days of gonadotrophin stimulation but the prognosis for these women remained poor, with clinical pregnancy rates ranging between 16% and 11% (5). We think that the choice of therapeutic protocol should aim to gain patient compliance and cost reduction in poor responder patients (17). A recent multicenter randomized trial demonstrated the non-inferiority of a mild ovarian stimulation strategy with a GnRH antagonist compared with a standard approach with a GnRH agonist. The ongoing pregnancy rate was 12.8% (25/195) for mild ovarian stimulation versus 13.6% (27/199) for conventional ovarian stimulation [95% confidence interval: (0.57-1.57)], and the duration of ovarian stimulation and amount of gonadotrophins used were significantly lower in the mild stimulation strategy (34).

Different studies performed on predicted poor responders showed that increasing FSH dose did not correlate with the number of retrieved oocytes (35). The maximum number of oocytes that could be retrieved in women was strongly limited by the number of recruitable antral follicles in the ovaries and a higher gonadotrophin dose was not able to compensate for the lack of substrate.

In conclusion, prediction of poor response can have positive results in terms of patient compliance and reduction of costs, but it does not seem to produce a significant improvement in IVF outcomes (36).

## PREDICTED HIGH RESPONSE

The term ‘hyper response’ refers to the retrieval of >15 oocytes (37) following a standard COS protocol. The prevalence rate in IVF cycles is estimated to be around 7% and decreases with the woman’s age. Young age, long menstrual cycles, polycystic ovary syndrome (PCOS), and hyper response in a previous cycle (38) are suggestive of high ovarian reserve, but the stronger predictors of hyper response are AMH and AFC. AMH cut-off levels proposed in literature for the prediction of hyper response vary according to the assay used (DSL, IBC or AMH gen II), but AMH serum levels >3.5 ng/mL have good sensitivity and specificity (2). An AFC value of >16 has been shown to be the most appropriate cut-off for hyper response (8, 39).

The measurement of ovarian reserve markers has a relevant value in patients with high ovarian reserve. First, it allows counselling couples about the potential risks associated with treatment, such as OHSS. Secondly, it permits choosing the treatment according to the predicted ovarian response. In patients with high ovarian reserve, COS individualization is crucial because it improves IVF outcomes and avoids the iatrogenic risk of OHSS. Recent studies demonstrated that the use of GnRH antagonists in predicted high responders was associated with a reduction in the incidence of OHSS. As a consequence, a reduction in cycle cancellation and patient hospitalization was achieved with a significant reduction in costs (17, 40). A large RCT including 1050 first IVF cycles recently demonstrated that the incidence of severe OHSS (5.1% vs. 8.9%; p=0.02) and moderate OHSS (10.2% vs. 15.6%; p=0.01) was significantly lower in the GnRH antagonist group compared with the agonist group, respectively, and pregnancy rates were similar in the two groups (40).

In GnRH antagonist protocols, initial follicular recruitment and selection is undertaken using endogenous endocrine factors prior to starting exogenous gonadotrophin administration. This leads to a lower number of growing follicles when compared with the standard long GnRH agonist protocol, which is why GnRH antagonist protocols are the first-choice treatment in women with high ovarian reserves at risk of OHSS. Secondly, GnRH antagonist protocols allow the possibility of inducing final oocyte maturation with an GnRH analogue instead of human chorionic gonadotrophin (hCG). This seems to significantly reduce the risk of OHSS, but it is associated with lower pregnancy rates in fresh IVF cycles because of an adverse effect on endometrium receptivity due to the absence of hCG (41). Implantation rates, clinical pregnancy rates, ongoing pregnancy rates, and survival rates of frozen-thawed embryos are similar in hCG with GnRH agonist trigger protocols, which demonstrates that GnRH agonist protocols do not impact on oocyte quality (42).

A strategy to improve outcomes in GnRH agonist-triggered cycles is the addition of a low dose (1500 IU) of hCG, administered 35 h or 5 days after the triggering bolus of GnRH agonist; however, this approach does not eliminate severe OHSS (23). Owing to the enhanced effectiveness of vitrification, segmentation in GnRH agonist-triggered cycles through the freezing of all embryos for transfer in subsequent cycles may be the optimal strategy to eliminate the risk of OHSS while maintaining elevated pregnancy rates (41, 43).

The FSH starting dose is another crucial determinant of ovarian response to stimulation. In women with high ovarian reserve, the choice of an unduly low gonadotrophin dose could lead to mono or pauci-follicular development. On the other hand, the choice of an excessive dose could lead to excessive ovarian response with subsequent OHSS risk. We believe that predictive algorithms based on reliable markers of ovarian reserve, such as those described above (25, 26), may guide physicians in this choice.

GnRH antagonists are better than GnRH-agonist in high responder patients at reducing the occurrence of OHSS, while maintaining comparable clinical pregnancy rates. Moreover, the FSH starting dose must be chosen on the basis of ovarian reserve markers.

## CONCLUSIONS

Ovarian reserve establishes prognosis in terms of oocyte retrieval and chances of live birth in IVF at any age of the woman. Medical history and a good assessment of ovarian reserve markers guarantee optimal oocyte retrieval, thereby formulating the most appropriate COS protocol for individual patients. The literature indicates how to guide correct management of patients, from predicted poor- to hyper-responders, but much remains to be done to reduce iatrogenic risks and improve IVF outcomes.
